# Pharmacokinetic incompatibility of the *Huanglian-Gancao* herb pair

**DOI:** 10.1186/s12906-020-2845-5

**Published:** 2020-02-22

**Authors:** Ji-Quan Zhang, Rui Wang, Ting Zhou, Qing Zhao, Chun-Cao Zhao, Bing-Liang Ma

**Affiliations:** 1 Engineering Research Center of Modern Preparation Technology of TCM of Ministry of Education, Shanghai University of Traditional Chinese Medicine, Shanghai, 201203 China; 2 Department of Pharmacology, School of Pharmacy, Shanghai University of Traditional Chinese Medicine, Cai Lun Road 1200, Shanghai, 201203 China

**Keywords:** Pharmacokinetic interaction, Herb-herb interaction, Experimental design, *Gancao*, *Huanglian*, Herb pair

## Abstract

**Background:**

Pharmacokinetic interaction is one of the most important indices for the evaluation of the compatibility of herbal medicines. Both *Gancao* (*Glycyrrhizae Radix* et *Rhizoma*) and *Huanglian* (*Coptidis Rhizoma*) are commonly used traditional Chinese medicines (TCMs). In this study, the influence of *Gancao* on the pharmacokinetics of *Huanglian* was systematically studied by using berberine as a pharmacokinetic marker.

**Methods:**

Extracts of the herbal pieces of *Huanglian* and the herb pair (*Huanglian* plus *Gancao*) were prepared with boiling water. The concentration of berberine in the samples was analyzed using liquid chromatography-mass spectrometry. The total amounts of berberine in all extract samples were compared. Comparative pharmacokinetic studies of *Huanglian* and the herb pair were conducted in ICR mice. In vitro berberine absorption and efflux were studied using mice gut sacs. The equilibrium solubility of berberine in the extracts was determined. The in vitro dissolution of berberine was comparatively studied using a rotating basket method.

**Results:**

*Gancao* significantly reduced berberine exposure in the portal circulation (425.8 ng·h/mL vs. 270.4 ng·h/mL) and the liver (29,500.8 ng·h/mL vs. 15,422.4 ng·h/mL) of the mice. In addition, *Gancao* decreased the peak concentration (C_max_) of berberine in the portal circulation (104.3 ng·h/mL vs. 76.5 ng·h/mL) and liver (4926.1 ng·h/mL vs. 2642.8 ng·h/mL) of mice. Significant influences of *Gancao* on the amount of berberine extracted (32% reduction), the solubility of berberine (34.7% compared with the control group), and dissolution (88.7% vs. 66.1% at 15 min in acid buffer and 68% vs. 51.8% at 15 min in phosphate buffer) were also revealed. Comparative pharmacokinetic studies in ICR mice indicated that the formation of sediment was unfavorable in terms of berberine absorption (345.3 ng·h/mL vs. 119.8 ng·h/mL).

**Conclusions:**

*Gancao* was able to reduce intestinal absorption and in vivo exposure of berberine in *Huanglian* via the formation of sediment, which caused reductions in the extracted amount, solubility, and dissolution of berberine.

## Background

Traditional Chinese medicine (TCM) is an ancient healing system that is guided by theories, such as Yin and Yang [1]. Herbal medicine is one of the main fields of TCM [1]. To ensure better curative efficacies and fewer side effects, multi-herb prescriptions, i.e., formulations which involve many herbs playing primary or secondary roles, are used as the conventional form of TCM [2]. Compatibility, which indicates interactions among the different herbal medicines, is an important property of a TCM formula [2]. The pharmacokinetic interactions among the different herbal medicines are one of the most important indices used to evaluate the compatibility of a TCM formula.

It is reported that some herbal medicines exhibit poor pharmacokinetic properties despite their excellent pharmacological effects [3]. The dissociation between the pharmacokinetic and pharmacological properties of herbal medicines is widely reported [3]. The underlying causes are associated with inconsistent drug exposure between the sites of action and systemic circulation [4]. Therefore, although there have been many studies on the pharmacokinetic compatibility of herbal medicines, the reliability of some previous studies is questionable as they have been limited to an evaluation of the influences of pharmacokinetic interactions on the exposure of the constituents of interest of herbal medicines in systemic circulation. In addition, in terms of the underlying causes of pharmacokinetic interactions, studies have focused predominantly on drug metabolic enzymes or transporters [5, 6]. Therefore, the experimental designs of some previous studies should be improved.

Herb pairs are important in the study of compatibility of herbal medicines [2]. *Gancao* (*Glycyrrhizae Radix* et *Rhizoma*) is prepared from the dried root and stem of plants, such as *Glycyrrhiza uralensis* Fisch., *Glycyrrhiza inflata* Bat., or *Glycyrrhiza glabra* L. [7]. Randomized controlled trials have revealed the efficacy of *Gancao* or its constituents for the treatment of asthma, liver diseases, and metabolic syndrome [8]. In addition, it is the most frequently used TCM owing to its “harmonic function”, which is supposed to improve the efficacy and/or reduce the toxicity of TCMs used concomitantly [8, 9]. *Huanglian* (*Coptidis Rhizoma*) is produced from plants, including *Coptis chinensis* Franch [9]. and has various pharmacological effects [10]. However, it causes acute toxicity [11]. Berberine is the major active constituent and a pharmacokinetic marker for *Huanglian* [10, 11]. It is a p-glycoprotein (P-gp) substrate [12] and is eliminated mostly via hepatic metabolism [13, 14]. The clinical efficacy of *Huanglian* or berberine for the treatment of diabetes and dyslipidemia has been proven [15, 16]. It is used together with *Gancao* in some famous TCM formulae, such as *Huanglian* decoction [17], *Gegen-qinlian* decoction [18], *Shengjiang Xiexin* decoction [19], and *Banxia Xiexin* decoction [20], which are recorded in the classical TCM book, *Shang Han Lun* (*Treatise on Cold Damage*) [21].

Both *Gancao* and *Huanglian* are used widely in the clinic; thus, research on their compatibility is helpful to guide their clinical applications. This aim of this study was to evaluate how *Gancao* influences the pharmacokinetics of *Huanglian* (by using berberine as a pharmacokinetic marker) in a systematic and integrated manner. In view of the irreplaceable role of animals in pharmacokinetic interaction studies, this study used ICR mice.

## Methods

### Plant material

Herbal specimens of *Huanglian* and *Gancao* were obtained from Shanghai Yang-he-tang Pharmaceutical Chain Co., Ltd. (Shanghai, China). The specimen numbers were No. 170531 and No. 170701, respectively. The herbs were identified as the root of *Coptis chinensis* Franch. and *Glycyrrhiza uralensis* Fisch., respectively. Authentication was performed by comparison with appropriate voucher specimens at the herbarium and by the analysis of the physical and chemical properties, according to *the Pharmacopoeia of People’s Republic of China* (2015 edition) [22].

### Chemicals and reagents

The reference standard (purity, > 98%) of berberine hydrochloride was purchased from the National Institute for the Control of Pharmaceutical and Biological Products (Beijing, China). Berberine hydrochloride (purity, > 95%) used for in vivo pharmacokinetic studies in mice was purchased from the Shanghai Yuan-Ye Biotechnology Co. Ltd. (Shanghai, China). The organic reagent acetonitrile was obtained from Merck (Darmstadt, Germany). All other materials were of analytical grade or higher.

### Preparation of the TCM extracts

The water extract of *Huanglian* (HLE) was prepared as described below. In brief, herbal pieces of *Huanglian* were extracted twice in ten volumes of boiling water (1.5 h for the first extraction and 1 h for the second extraction). The obtained extract was then filtered through eight layers of gauze and vacuum dried at a low temperature (60 °C). For the herb pair, herb pieces of *Huanglian* were extracted along with an equal weight of *Gancao* (HL-GCE). The ratio of *Huanglian* and *Gancao* (1,1) was chosen to match the ratio in the *Huanglian* Decoction.

### Quality control of the TCM extracts

Several constituents of the TCM extracts were measured using an LC-MS system [HPLC (Shimadzu, UFLC-XR system, Japan) with a single-stage mass spectrometer [Thermo Fisher Scientific, (LCQ Ion trap, Bremen, Germany)]. Carbamazepine and mangiferin were used as the positive and negative internal standards, respectively. The extracts were dissolved in 50% methanol. After centrifugation (16,000 rpm, 4 °C, 10 min), the supernatant was collected and 10 μL of the supernatant was injected into the LC-MS system. An Agilent Eclipse XDB-C18 column (100 × 4.6 mm, 5 μm) was used for chromatographic separation of the analytes. The column temperature was maintained at 30 °C. Water containing acetic acid (0.1%) and ammonium acetate (5 mM) was used as the mobile phase A, and acetonitrile was used as the mobile phase B. The following elution gradient was used: 0–1 min, 10% B; 1–5 min, 10–40% B; 5–11 min, 40–60% B; 11–24 min, 60–90% B; 24–26 min, 90% B; and 26.1–28 min, 10% B. The flow rate was 1.0 mL/min. Both positive and negative full-scans (mass range, 100–1000 m/z) were performed. In negative ionization mode, deprotonated [M − H]^−^ molecular ions of glycyrrhizic acid (m/z 821), liquiritin (m/z 417), and mangiferin (m/z 421) were generated. In positive ionization mode, protonated [M + H]^+^ ions of berberine (m/z 336), coptisine (m/z 320), jatrorrhizine (m/z 338), palmatine (m/z 352), epiberberine (m/z 336), and carbamazepine (m/z 237) were generated. The linear dynamic range for glycyrrhizic acid and liquiritin was 3.125 to 200 μg/mL. The linear dynamic range for berberine, coptisine, jatrorrhizine, palmatine, and epiberberine was 0.156 to 10 μg/mL. The method was validated in terms of accuracy, precision, recovery, repeatability, and stability (Data not Shown).

After the aqueous extract of *Huanglian* was obtained, the concentration of berberine in the liquid extract was estimated, and the volume of the liquid extract was recorded. The total amount of extracted berberine was obtained by measuring the concentration of berberine and the volume of the liquid extract.

The mass percentages of berberine, jatrorrhizine, palmatine, coptisine, and epiberberine in dried HLE were measured as 22.0, 4.8, 2.5, 5.0, and 6.2%, respectively. The mass percentages of the representative alkaloids in *Huanglian* (berberine, coptisine, jatrorrhizine, palmatine, and epiberberine) and the mass percentages of the representative constituents of *Gancao* (glycyrrhizic acid and liquiritin) in dried HL-GCE were determined to be 6.6, 1.4, 0.8, 1.8, 1.7, 0.46, and 0.44%, respectively.

### Quantitative analysis of the biological samples

The concentration of berberine in plasma and liver homogenate was analyzed by a liquid chromatography-tandem mass spectrometry (LC-MS/MS) system using the validated method employed in our published studies [23, 24]. The HPLC instrument used was made by Shimadzu (Shimadzu Prominence UFLC-XR series, Japan), and the mass spectrometer used was made by Thermo Scientific (Thermo Scientific TSQ Quantum Ultra, Waltham, MA, USA). An electrospray ionization (ESI) source was used. The internal standard used was carbamazepine. The biological samples were precipitated in three volumes of acetonitrile. After centrifugation at 16,000 rpm for 10 min at 4 °C, an equal volume of water was completely mixed with the obtained supernatant. A 10 μL volume of this solution was added to a C18 analytical column (Hypersil Gold, 5 μm, 100 × 2.1 mm) that was used for the chromatographic separation of the analytes. The mobile phase was composed of solvent A [formic acid (0.08% v/v) and ammonium acetate (2 mM) in water] and solvent B (acetonitrile). The gradient elution was as follows: 0–7 min, 15%–68% B; 7.01–10 min, 15–15% B. The flow rate was 0.3 mL/min. The positive ion mode was used for the ESI source and data acquisition was performed in the multiple reaction monitoring (MRM) mode. Quantification was performed using the transitions *m/z* 336.2 → 322.3 and *m/z* 237.0 → 194.3 for berberine and carbamazepine, respectively. The linear range of berberine was 1.95–1000 ng/mL.

### Experimental animals

ICR mice (male and female, Grade II, 24 ± 2 g body weight) were obtained from Shanghai Slac Laboratory Animal Co., Ltd. (Shanghai, China). Before the experiment, the mice were acclimated for at least 3 days in an air-conditioned room at 22 °C–24 °C, under a 12-h dark/light cycle and given food and water ad libitum. Before the pharmacokinetic experiments, the mice were fasted for approximately 12 h, although they were provided with drinking water ad libitum. All animal experimental protocols were approved (PZSHUTCM19011105) and all experiments were performed in accordance with the guidelines of the Institutional Animal Care and Use Committee of Shanghai University of Traditional Chinese Medicine.

### Pharmacokinetic studies in mice

For the comparative pharmacokinetic study of HLE and HL-GCE, approximately 84 ICR mice were used, and they were randomly divided into two groups. The mice received 0.88 g/kg oral HLE (that is, the dose of herbal pieces of *Huanglian* was 3 g/kg and the dose of berberine was 194 mg/kg) or 2.00 g/kg HL-GCE (that is, the dose of herbal pieces of *Huanglian* was equal to that used in the HLE-treated groups, i.e., 3 g/kg, and the dose of berberine was 132 mg/kg). The dose of HLE, which was equivalent to the clinical dose of the herb pieces of *Huanglian* of approximately 0.3 g/kg per day for adults, was chosen based on its clinical applications, i.e., 15–45 g per day for adults [25]. In addition, the dose of berberine in HLE was consistent with its clinical applications [26] and preclinical studies in mice, i.e., 0.1–0.3 g/kg/day [27].

For the comparative study of the supernatant and the sediment of the water solution of HL-GCE, which were obtained by centrifuging the water solution of HL-GCE at 16,000 rpm for 10 min, approximately 84 ICR mice were used and were randomly divided into two groups. The mice were orally administered the supernatant or sediment, which contained equal amounts of berberine (200 mg/kg).

Six mice in each group were anesthetized using diethyl ether at 0.25, 0.5, 1, 2, 4, 8, or 12 h after administration, and blood samples were obtained from the portal circulation and posterior orbital venous plexus, respectively. Heparin was used as the anticoagulant. The plasma samples were obtained by centrifugation of blood samples at 3000 rpm for 10 min at 4 °C. The liver tissues of the mice were collected and then homogenized in ten volumes of water. At the end of the experiment, the mice were euthanized by cervical dislocation. The plasma and liver homogenate samples were stored at − 80 °C. The concentration of berberine in each sample was then determined after appropriate dilution using LC-MS.

### In vitro absorption and efflux of berberine in HLE or HL-GCE

Approximately 24 ICR mice were used in this experiment and were randomly divided into six groups. After the mice were euthanized by dislocation of the cervical vertebrae, a laparotomy was performed, and a segment of the ileum approximately 12 cm long was removed. The ileum segment was washed with chilled Krebs-Ringer buffer (118 mM NaCl, 25 mM NaHCO_3_, 1.2 mM MgSO_4_, 2.5 mM CaCl_2_, 11 mM glucose, 1.2 mM KH_2_PO_4_, and 4.7 mM KCl, pH 6.8), and then ligated at one end. To study the intestinal absorption of berberine, the gut sac was filled on the inner mucosal side with 1 mL of Krebs-Ringer buffer containing HLE (200 μg/mL or 10 mg/mL berberine) or HL-GCE with the corresponding concentration of berberine. For the study of berberine efflux, the gut sac was everted and filled on the inner serosal side with 1 mL Krebs-Ringer buffer containing HLE (200 μg/mL berberine) or HL-GCE with the corresponding concentration of berberine. The other end of the ileum was then tightly ligated. The sac was then incubated at 37 °C in a Magnus bath containing 20 mL of blank Krebs-Ringer buffer. Aliquots of buffer (100 μL) were sampled from the Magnus bath at every 15 to 60 min of incubation. Blank Krebs-Ringer buffer at an equal volume was immediately added. The concentrations of berberine in the obtained samples were determined and normalized by using the lengths of the sacs that were measured after incubation.

### Equilibrium solubility of berberine in HLE or HL-GCE

The equilibrium solubility of berberine was studied in HLE or HL-GCE. In brief, 1 mL water was added to various amounts of HLE (18.75, 37.5, 75, 150, or 300 mg, the amounts of berberine were 4.125, 8.25, 16.5, 33, or 66 mg, respectively) or HL-GCE (containing 4.125, 8.25, 16.5, 33, or 66 mg berberine). The concentrations of HLE were chosen in accordance with concentrations reported in the studies of pharmacokinetics (18.75, 37.5, or 75 mg/mL) or acute toxicity (150, or 300 mg/mL) [28]. The suspensions were treated with ultrasound (1 h) and then kept undisturbed at room temperature (approximately 22 °C) for 5 h. After centrifugation at 16,000 rpm for 10 min, the supernatant was obtained. The concentration of berberine in each sample was then determined after appropriate dilution using the LC-MS method.

### In vitro dissolution of berberine in HLE or HL-GCE

The experiments were performed using an RC-MD dissolution tester (Tianda Tianfa Technology Co., Ltd., Tianjin, China) through a rotating basket method. The rotational speed of the basket was 100 rpm. The maintained temperature of the dissolution medium was 37 °C ± 0.5 °C. The extracts containing 10 mg berberine were enclosed in blank capsules and placed in the baskets. Then, the baskets were placed into the dissolution vessel containing 900 mL of hydrochloric acid buffer (pH 1.2), mimicking the gastric fluid or 900 mL of phosphate buffer (0.2 mol/L sodium phosphate, pH 6.8), mimicking the intestinal juices. To study the hydrochloric acid buffer, sampling was performed at 2.5, 5, 10, 15, 20, 30, 45, 60, and 90 min. For the experiments in the phosphate buffer, sampling was performed at 2.5, 5, 15, 30 min, and 1, 2, 3, and 4 h. The withdrawn slurry was filtered using 0.22 μm cellulose filters. After centrifugation (16,000 rpm, 10 min), the concentration of berberine in each sample was determined using LC-MS.

### Statistical analyses

Pharmacokinetic parameters were calculated by non-compartmental analysis (NCA) using Phoenix WinNonlin (version 6.1, Pharsight Corporation, CA, USA). The results were expressed as mean ± standard deviation (SD). For multiple comparisons, one-way or two-way analysis of variance (ANOVA) was performed (^*^indicated *P* < 0.05, ^**^indicated *P* < 0.01).

## Results

### Total amount of extracted berberine in HLE and HL-GCE

In total, 16.15 g berberine was extracted from 250 g herbal pieces of *Huanglian*. The presence of 250 g herbal pieces of *Gancao* reduced around 32% of the extracted amount of berberine to 10.98 g.

### Pharmacokinetics of berberine in HLE and HL-GCE

**Fig. 1:**
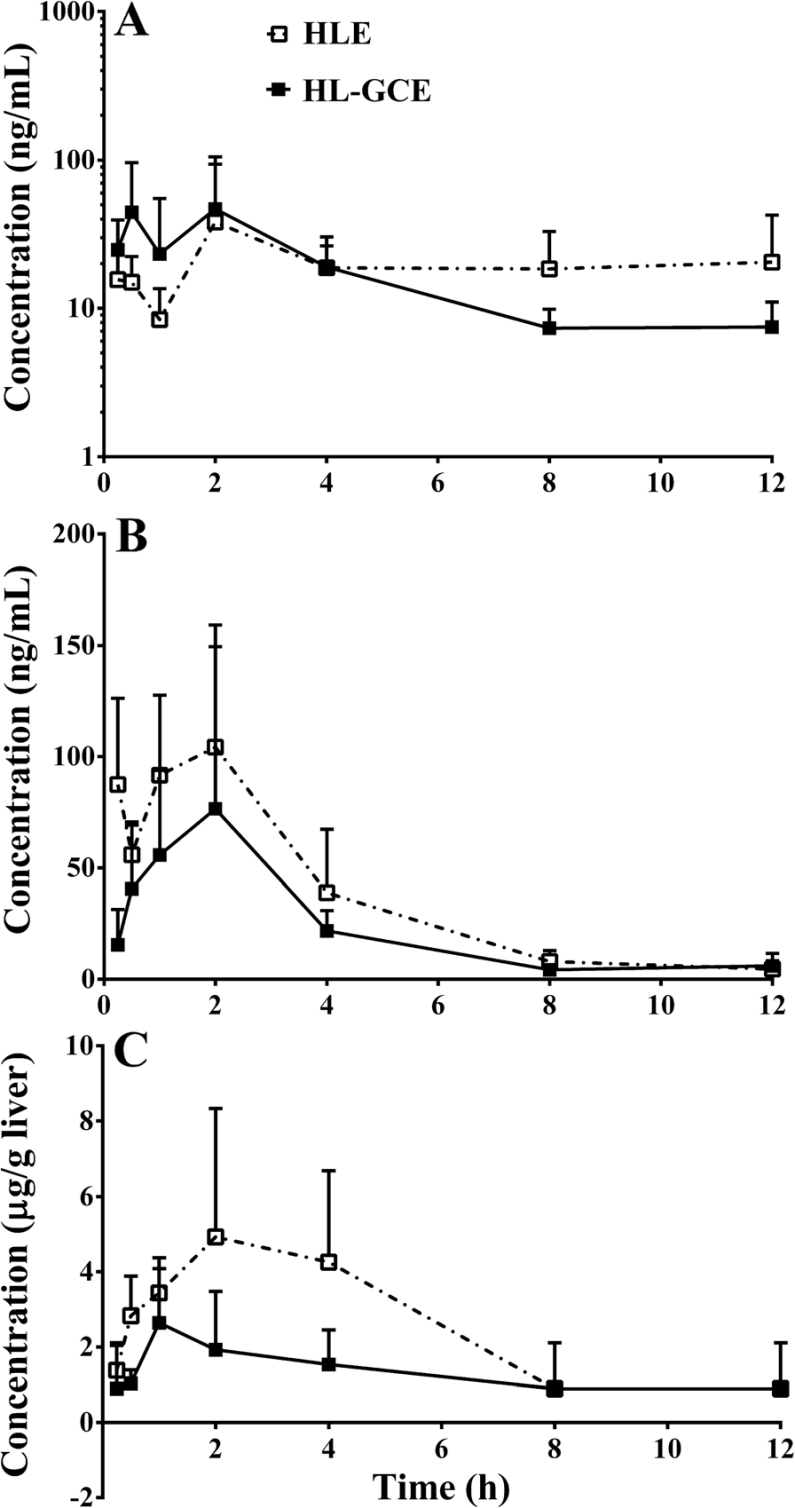
Concentration-time curves of berberine in blood samples from the systemic circulation (**a**), portal vein (**b**), and the liver (**c**) of mice that received 0.88 g/kg oral *Huanglian* extract (HLE, 194 mg/kg berberine accordingly) or 2.00 g/kg *Huanglian*-*Gancao* extract (HL-GCE, 132 mg/kg berberine accordingly) (mean ± SD, *n* = 6). The HLE- and HL-GCE-treated groups were administered equal doses of the herb pieces of *Huanglian* (3 g/kg)

The berberine time curves in mice that received oral HLE or HL-GCE, which were equal in terms of the dose of the herbal pieces of *Huanglian* (3 g/kg), are shown in Fig. 1. The detailed pharmacokinetic parameters are shown in Table 1. The results showed that *Gancao* did not significantly influence the exposure (AUC_0–12 h_) of berberine in systemic circulation (244.2 ng·h/mL vs. 211.8 ng·h/mL, *P* > 0.05), but significantly reduced berberine by 36.5% in the portal circulation (425.8 ng·h/mL vs. 270.4 ng·h/mL, *P* < 0.01) and by 48.7% in the liver (29,500.8 ng·h/mL vs. 15,422.4 ng·h/mL, *P* < 0.01), respectively. In addition, *Gancao* decreased the peak concentration (C_max_) of berberine in the portal circulation (104.3 ng·h/mL vs 76.5 ng·h/mL) and the liver (4926.1 ng·h/mL vs. 2642.8 ng·h/mL) of mice.

**Table 1:** Pharmacokinetic parameters of berberine in *Huanglian* and *Huanglian*-*Gancao* herb pair treated mice (mean ± SD, *n* = 6)

Samples	Groups	T_1/2_ (h)	T_max_ (h)	C_max_ (ng/mL)	AUC_0-t_ (ng·h/mL)	AUC_0-∞_ (ng·h/mL)	MRT_0-t_ (h)
Systemic circulation	HL	14.9	2.0	38.2	244.2	623.8	5.9
	HL + Gancao	3.8	2.0	46.8	211.8	242.4	3.8
Portal circulation	HL	2.6	2.0	104.3	425.8	440.3	2.9
	HL + Gancao	4.3	2.0	76.5	270.4	297.7	3.1
Liver	HL	3.5	2.0	4926.1	29,500.8	33,013.3	4.0
	HL + Gancao	8.5	1.0	2642.8	15,422.4	25,088.6	4.9

AUC, the area under the concentration time curve; C_max_, peak concentration; HL, Huanglian; MRT, mean retention time; T_1/2_, elimination half-life; T_max_, time to reach peak concentration

### Pharmacokinetics of the supernatant and sediment in the water solution of HL-GCE

**Fig. 2:**
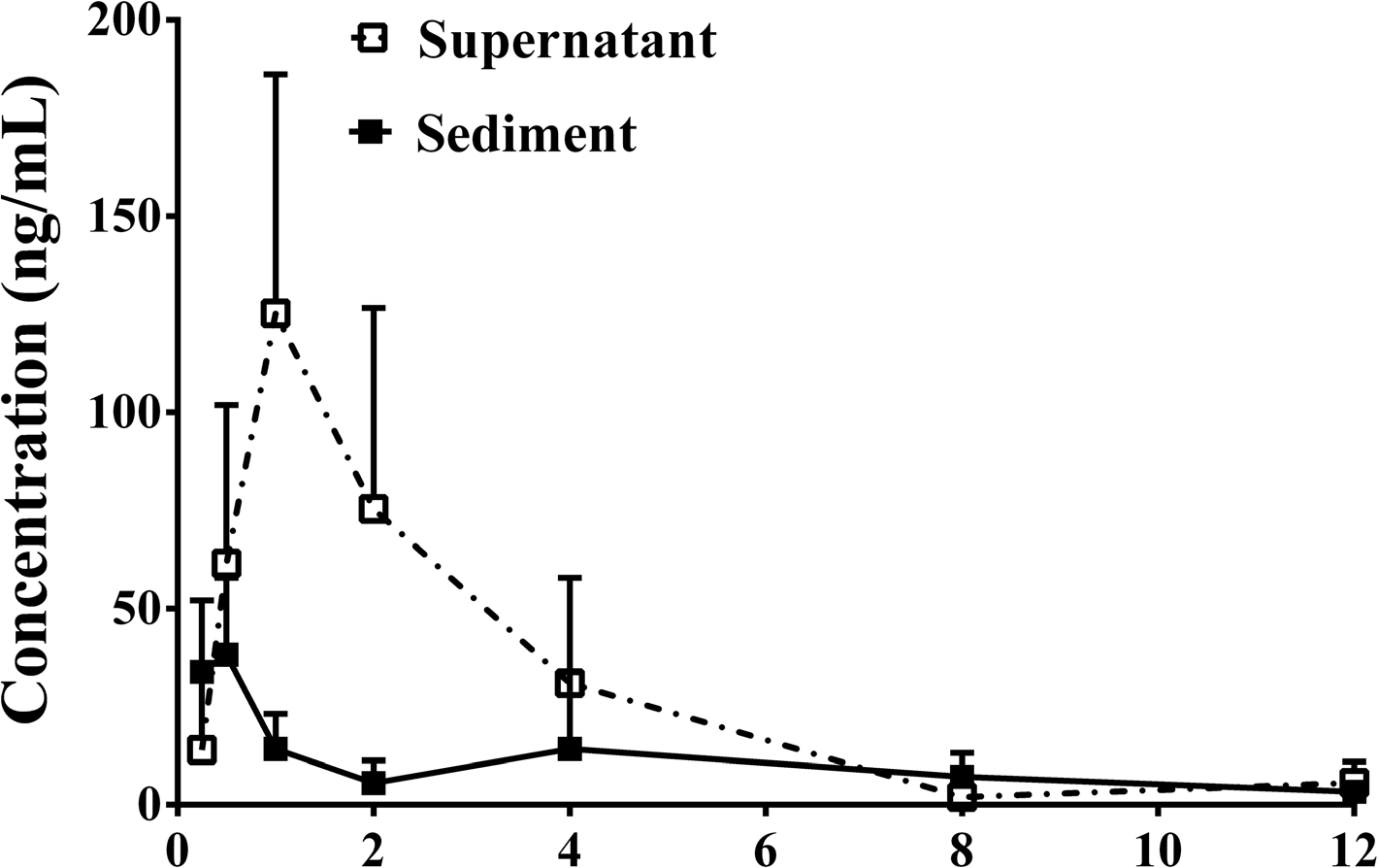
Concentration-time curves of berberine in the portal vein of mice that received the supernatant or sediment of the water solution of *Huanglian*-*Gancao* extract (HL-GCE) (mean ± SD, *n* = 6). The supernatant- and sediment-treated groups received equal doses of berberine (200 mg/kg)

As shown in Fig. 2, the exposure (AUC_0–12 h_) of berberine in the portal circulation of mice that received the supernatant of the water solution of HL-GCE was approximately 2.88 times higher than that in the portal circulation of sediment-treated mice (345.3 ng·h/mL vs. 119.8 ng·h/mL, *P* < 0.01), indicating that the formation of sediment was unfavorable in terms of berberine absorption.

### Intestinal absorption and efflux of berberine in HLE and HL-GCE

**Fig. 3:**
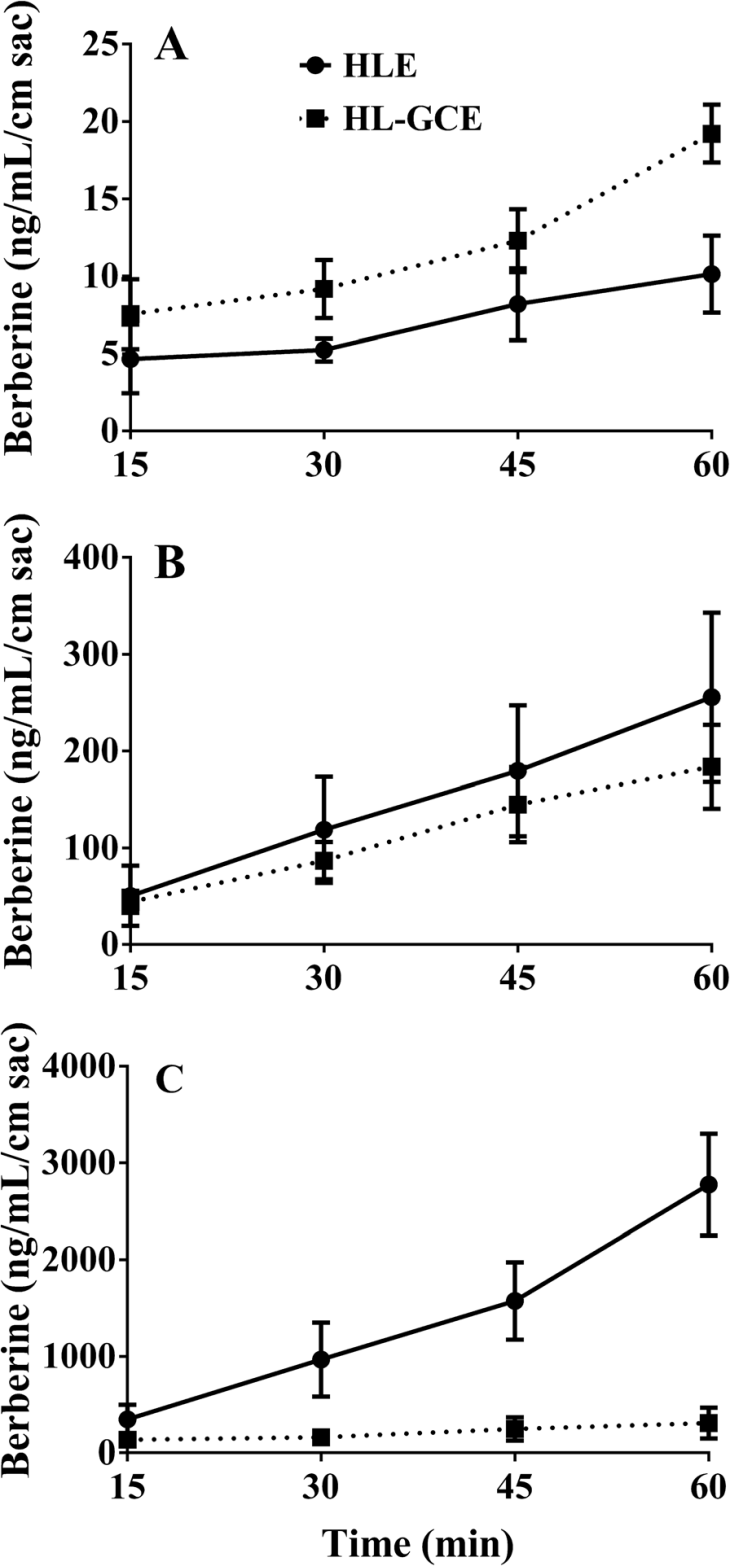
Intestinal absorption and efflux of berberine in the *Huanglian* extract (HLE) or the *Huanglian-Gancao* extract (HL-GCE) (mean ± SD, *n* = 6). **a**, Absorption of berberine at a low concentration (200 μg/mL); **b**, Efflux of berberine at a low concentration (200 μg/mL); **c**, Absorption of berberine at a high concentration (10 mg/mL)

As shown in Fig. 3, when berberine was present at a low concentration (200 μg/mL) and therefore completely dissolved, the presence of *Gancao* significantly increased the absorption of berberine (*P* < 0.01, A) and decreased its efflux in the gut sac of mice (*P* < 0.05, B). In contrast, when berberine was present at a high concentration (10 mg/mL, not completely dissolved), which was consistent with the concentration used in the pharmacokinetic studies (i.e., 10 mg/mL), *Gancao* significantly reduced the intestinal absorption of berberine (*P* < 0.01).

### Equilibrium solubility of berberine in HLE or HL-GCE

**Fig. 4:**
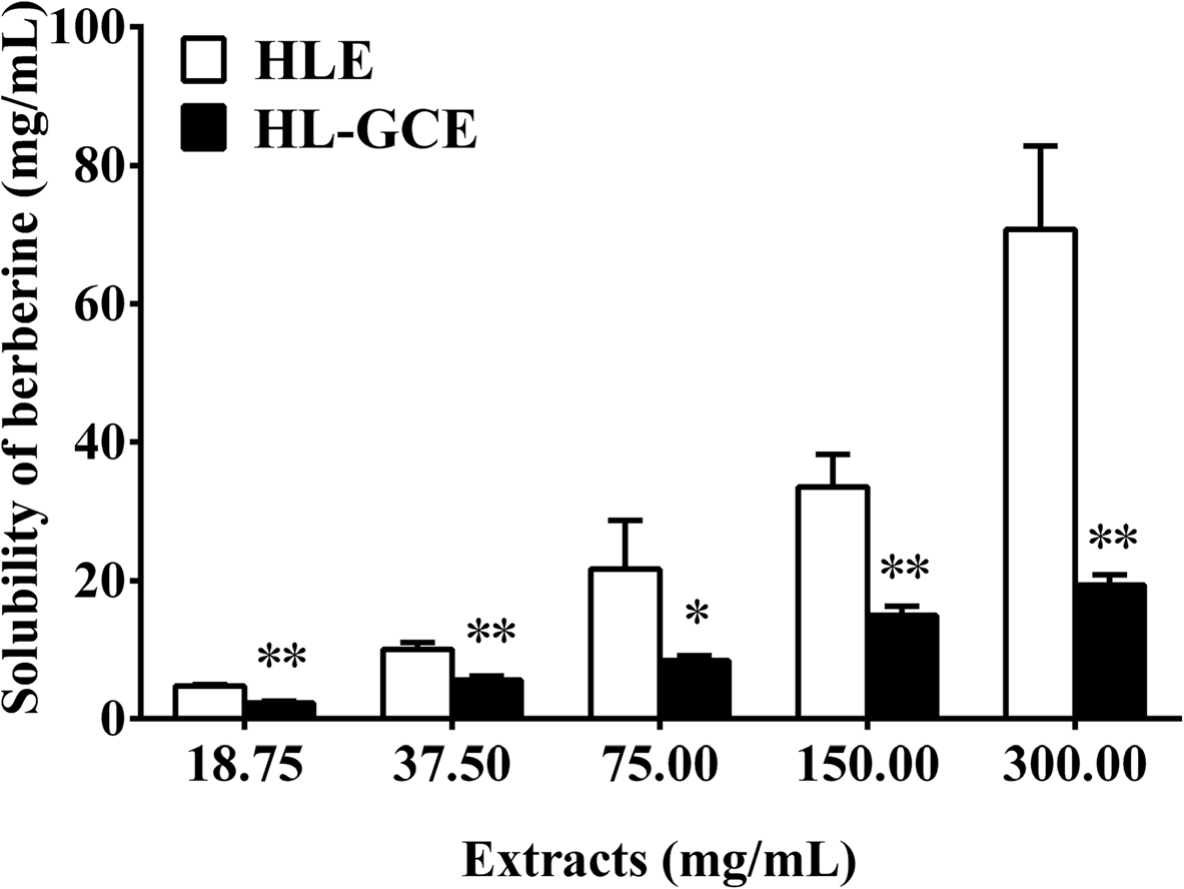
Water solubility of berberine in *Huanglian* extract (HLE) or *Huanglian*-*Gancao* extract (HL-GCE) (mean ± SD, *n* = 3). *, *P* < 0.05, **, *P* < 0.01 compared with the HLE group

As shown in Fig. 4, berberine was fully dissolved in each HLE group. In contrast, the equilibrium solubility of berberine in the HL-GCE groups was significantly lower than that in the corresponding HLE groups (*P* < 0.01). With an increase in the extract concentrations from 18.75 to 300 mg/mL, the solubility of berberine in the HL-GCE groups was 55.5, 60.9, 46.6, 52.6, and 34.7% of those in the corresponding HLE groups.

### In vitro dissolution of berberine

**Fig. 5:**
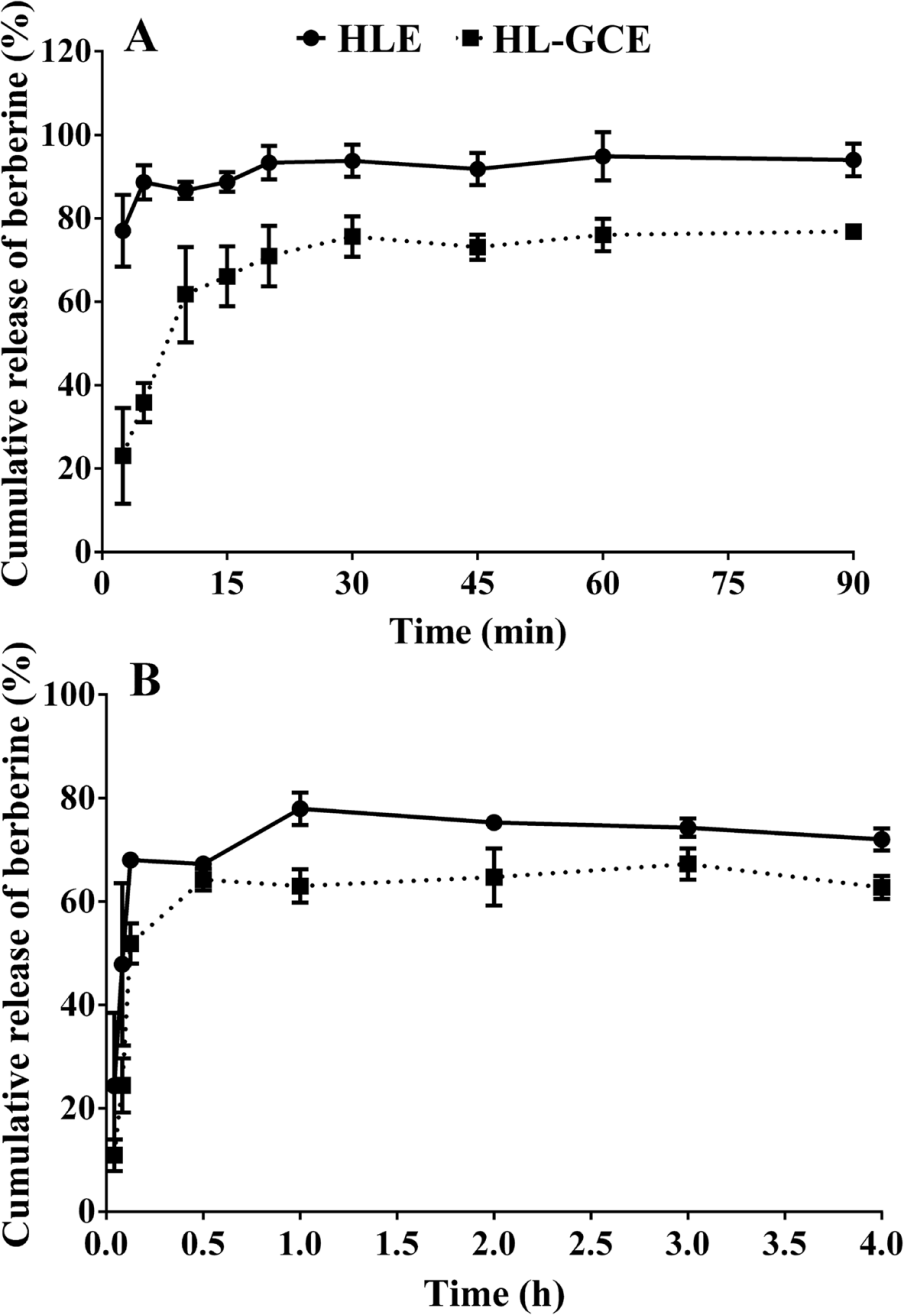
Powder dissolution profile of berberine in *Huanglian* extract (HLE) and *Huanglian*-*Gancao* extract (HL-GCE) in pH 1.2 hydrochloric acid buffer (**a**) and pH 6.8 phosphate buffer (**b**) (mean ± SD, *n* = 3)

As shown in Fig. 5, berberine in HLE had a higher dissolution rate than that in HL-GCE. After incubation for 15 min, approximately 88.7 and 68% of berberine in HLE were released into the hydrochloric acid buffer (A) and phosphate buffer (B), respectively. However, the release of berberine in HL-GCE was 66.1 and 51.8%, respectively, for hydrochloric acid buffer (A) and phosphate buffer (B).

## Discussion

The results revealed that the addition of *Gancao* caused inconsistent changes in the berberine exposure level in the systemic circulation and those in the portal circulation and the liver. Thus, there was an indistinctive influence in the systemic circulation but the berberine exposure level was significantly reduced in the portal circulation and the liver. In other words, these results, for the first time, verified the limitation of determining the pharmacokinetic herb-herb interactions based on the dynamic changes of drug levels solely in the systemic circulation. Therefore, the pharmacokinetic compatibility of TCM formulations should be systematically studied. If necessary, the influences of drug levels at other in vivo sites, especially the target tissues of drug action, should be investigated. The mechanisms mediating the inconsistent changes may be related to active drug transport and differences in protein binding in various fluids or tissues [4] that remained to be studied.

Based on the results in the liver tissues, it can be concluded that *Gancao* significantly decreased the in vivo exposure of berberine in *Huanglian*. Furthermore, based on the results regarding the portal circulation, it could be concluded that the effect of *Gancao* was mainly due to a reduction in the intestinal absorption of berberine. However, the underlying mechanisms were complicated and should be explained in an integrative manner.

*Gancao* exerted a profound adverse impact on the solubility of berberine owing to the formation of sediment, which was unfavorable in terms of the intestinal absorption of berberine. When *Gancao* and *Huanglian* were extracted together, complexes of saponins and berberine (or its homolog, epiberberine) were produced [29]. In the generated complexes, the hydrophilic carboxylic groups of the saponins were associated with the quaternary ammonium ions in the alkaloid, and they were thus retained inside the complex, with the hydrophobic part of the saponins exposed to the aqueous phase, thereby forming sediments [29]. Furthermore, the results showed that *Gancao* reduced berberine release from the extract. In addition, given the negative effects on the extracted amount of berberine, *Gancao* decreased the oral amount of berberine when administered at the same dose as that of the herbal pieces of *Huanglian*. However, when berberine was entirely dissolved at a low concentration, *Gancao* improved berberine absorption via the inhibition of its efflux; that is, *Gancao* increased the permeation of berberine. Our results were in accordance with those of the previous studies involving a single-pass intestinal perfusion model rat with jugular vein cannulation [30]. During intestinal absorption, berberine is extensively pumped out by transporters, such as P-gp [31]. Consequently, inhibition of P-gp by glycyrrhizic acid, a major constituent of *Gancao*, improved the in vitro permeability of berberine in a Caco-2 cell monolayer and increased its in vivo exposure in rats by approximately six-fold [18]. It should be pointed out that the influences of *Gancao* on the intestinal metabolism of berberine were not examined in this study on the assumption that the induction of drug-metabolizing enzymes that is supposed to decrease the intestinal absorption of berberine would not occur unless there is a sustained stimulus [5]. In brief, although *Gancao* improved the intestinal permeability of berberine, it ultimately led to a major decrease in the in vivo exposure of berberine owing to reductions in its extracted amount, solubility, and dissolution from the TCM extract. In contrast to the commonly concerned drug transporters and metabolizing enzymes, the results highlighted the crucial roles of solubility, extracted amount, and dissolution behavior of constituents in pharmacokinetic herb-herb interactions. However, it should be noted that our results did not exclude the significance of drug metabolic enzymes and transporters in pharmacokinetic herb-herb interactions. The mechanisms of pharmacokinetic interactions should better be studied on a case to case basis.

Gut sacs have been used widely to study the intestinal absorption of drugs [32]. However, the concentration of the compound of interest should be carefully chosen. Our results revealed the concentration-dependent pharmacokinetic interactions between the *Huanglian-Gancao* herb pair in terms of intestinal absorption. Using a relatively low berberine concentration in the gut sac experiment, we were only able to elucidate the influence on drug permeability. However, when the concentration of berberine used was similar to that used in pharmacokinetic studies, we were able to demonstrate the integrated influences on the solubility, release behavior, and intestinal permeability of the compound of interest.

Experimental animals were fully utilized in this study, that is, multiple samples, such as systemic circulation blood, portal vein blood, and tissues (the liver in this study), were simultaneously collected from one animal. However, it should be indicated that this study has some limitations. Considering the species difference, the pharmacokinetic interaction between *Huanglian* and *Gancao* should be confirmed in humans.

## Conclusions

In summary, our results showed that *Gancao* was able to reduce the intestinal absorption of berberine in *Huanglian* through reductions in the extracted amount, solubility, and release from the herb extract. The study revealed the limitations of determining the pharmacokinetic herb-herb interactions based solely on evaluating drug levels in the systemic circulation. In addition, the study proposed a simple method to predict pharmacokinetic herb-herb interactions based on gut sac studies using an in vivo pharmacokinetic study relevant concentration. We believe that this study will have a significant impact on future studies on pharmacokinetic herb-herb interactions.

## Supplementary information

**Additional file 1.** Supplementary Figure 1. Total ion chromatogram of Huanglian extract.

**Additional file 2.** Supplementary Figure 2. Extracted ion chromatogram of berberine in Huanglian extract.

## Data Availability

The datasets used and/or analysed during the current study are available from the corresponding author on reasonable request.

## Abbreviations

ANOVA: analysis of variance
AUC: area under the curve
ESI: electrospray ionization
HLE: *Huanglian* extract
HL-GCE: *Huanglian*-*Gancao* extract
HPLC: high performance liquid chromatography
LC-MS/MS: liquid chromatography tandem mass spectrometry
m/z: mass-to-charge ratio
MRM: multiple reaction monitoring
MS: mass spectrometry
P-gp: p-glycoprotein
SD: standard deviation
TCM: traditional Chinese medicine
