# Supplementary figures and images for: Pharmacokinetic incompatibility of the Huanglian-Gancao herb pair
Source: BMC Complement Med Ther. 2020 Feb 22;20:61. doi: 10.1186/s12906-020-2845-5 (PMC7076871; doi:10.1186/s12906-020-2845-5)

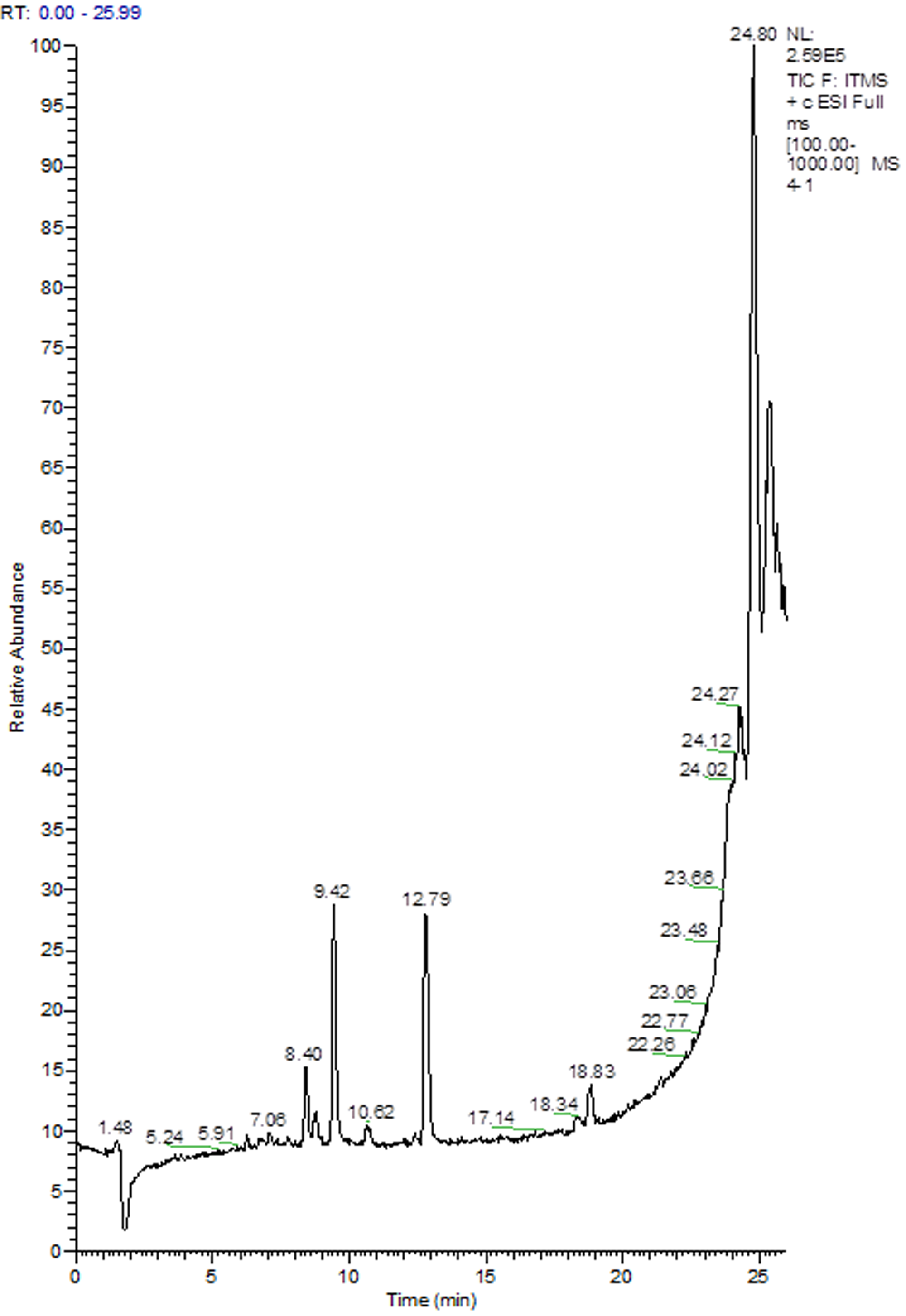

Supplement: Supplementary file 1 — Additional file 1. Supplementary Figure 1. Total ion chromatogram of Huanglian extract. [file 12906_2020_2845_MOESM1_ESM.tif]

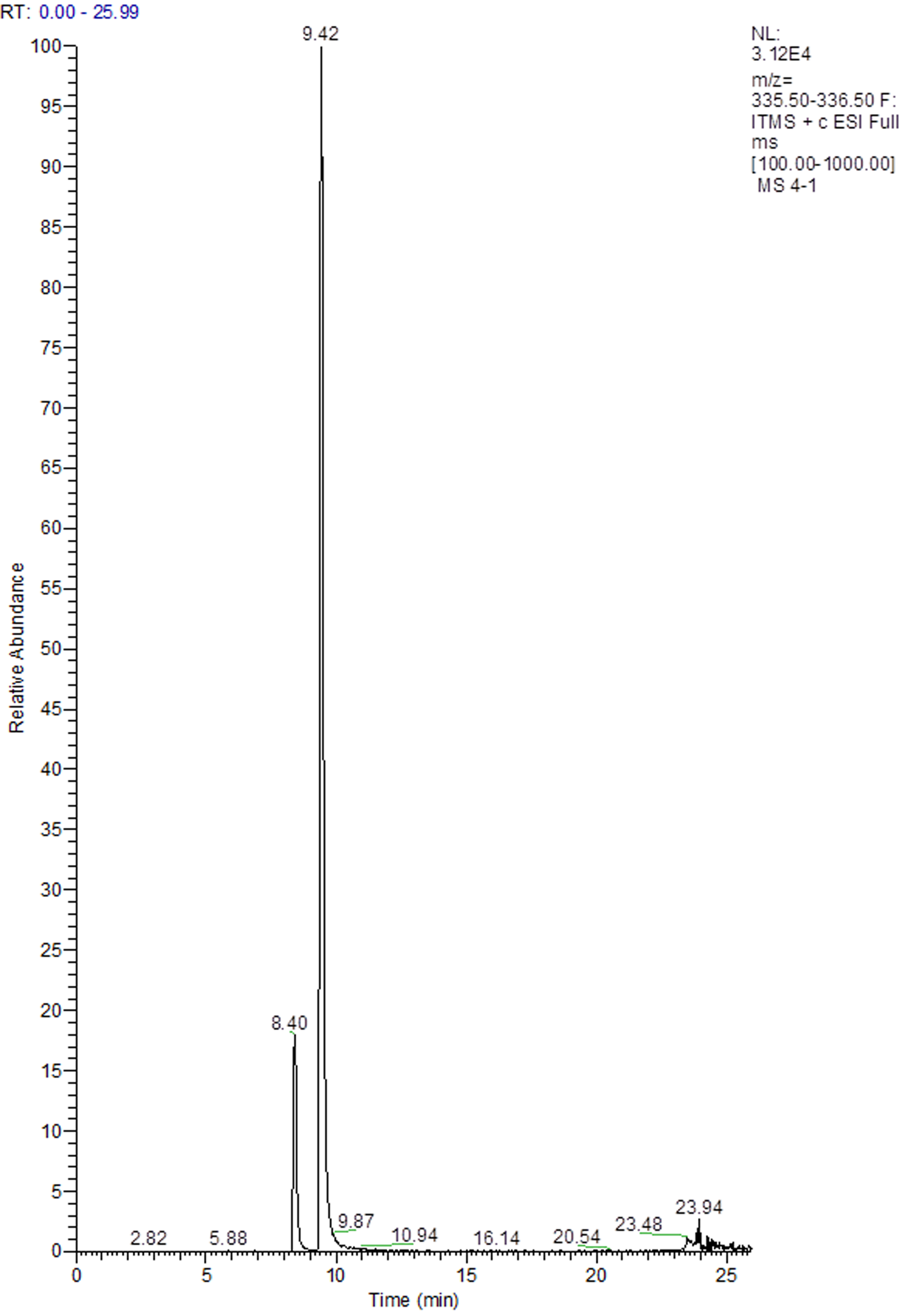

Supplement: Supplementary file 2 — Additional file 2. Supplementary Figure 2. Extracted ion chromatogram of berberine in Huanglian extract. [file 12906_2020_2845_MOESM2_ESM.tif]
